# SOX9 is a dose-dependent metastatic fate determinant in melanoma

**DOI:** 10.1186/s13046-018-0998-6

**Published:** 2019-01-14

**Authors:** Xintao Yang, Rui Liang, Chunxi Liu, Jessica Aijia Liu, May Pui Lai Cheung, Xuelai Liu, On Ying Man, Xin-Yuan Guan, Hong Lok Lung, Martin Cheung

**Affiliations:** 1 School of Biomedical Sciences, Li Ka Shing Faculty of Medicine, The University of Hong Kong, 21 Sassoon Road, Hong Kong, China; 2 Department of Anesthesiology, Zhejiang Cancer Hospital, Hangzhou, Zhejiang China; 3 Department of Pediatric Surgery, Second Hospital of Hebei Medical University, Shijiazhuang, Hebei China; 4 Department of Biology, Faculty of Science, Hong Kong Baptist University, Hong Kong, China; 5 Department of Clinical Oncology, Li Ka Shing Faculty of Medicine, The University of Hong Kong, Hong Kong, China

**Keywords:** SOX9; SOX10, NEDD9, RHOA, RAC1, Melanoma, Metastatic, p21

## Abstract

**Background:**

In this research, we aimed to resolve contradictory results whether SOX9 plays a positive or negative role in melanoma progression and determine whether SOX9 and its closely related member SOX10 share the same or distinct targets in mediating their functions in melanoma.

**Methods:**

Immunofluorescence, TCGA database and qPCR were used to analyze the correlation between the expression patterns and levels of SOX9, SOX10 and NEDD9 in melanoma patient samples. AlamarBlue, transwell invasion and colony formation assays in melanoma cell lines were conducted to investigate the epistatic relationship between SOX10 and NEDD9, as well as the effects of graded SOX9 expression levels. Lung metastasis was determined by tail vein injection assay. Live cell imaging was conducted to monitor dynamics of melanoma migratory behavior. RHOA and RAC1 activation assays measured the activity of Rho GTPases.

**Results:**

High SOX9 expression was predominantly detected in patients with distant melanoma metastases whereas SOX10 was present in the different stages of melanoma. Both SOX9 and SOX10 exhibited distinct but overlapping expression patterns with metastatic marker NEDD9. Accordingly, SOX10 was required for NEDD9 expression, which partly mediated its oncogenic functions in melanoma cells. Compensatory upregulation of SOX9 expression in SOX10-inhibited melanoma cells reduced growth and migratory capacity, partly due to elevated expression of cyclin-dependent kinase inhibitor p21 and lack of NEDD9 induction. Conversely, opposite phenomenon was observed when SOX9 expression was further elevated to a range of high *SOX9* expression levels in metastatic melanoma specimens, and that high levels of SOX9 can restore melanoma progression in the absence of SOX10 both in vitro and in vivo. In addition, overexpression of SOX9 can also promote invasiveness of the parental melanoma cells by modulating the expression of various matrix metalloproteinases. SOX10 or high SOX9 expression regulates melanoma mesenchymal migration through the NEDD9-mediated focal adhesion dynamics and Rho GTPase signaling.

**Conclusions:**

These results unravel NEDD9 as a common target for SOX10 or high SOX9 to partly mediate their oncogenic events, and most importantly, reconcile previous discrepancies that suboptimal level of SOX9 expression is anti-metastatic whereas high level of SOX9 is metastatic in a heterogeneous population of melanoma.

**Electronic supplementary material:**

The online version of this article (10.1186/s13046-018-0998-6) contains supplementary material, which is available to authorized users.

## Background

Melanoma is one of the most devastating types of human cancer and is the main cause of skin cancer deaths [1]. The aggressiveness of melanoma is due to the combined effects of oncogenic signaling pathways and cancer-relevant transcription factors, resulting in the transformation of neural crest (NC)-derived pigment cells (melanocytes) located in the basal layer of the skin into metastatic melanoma. Melanoma is surgically curable when diagnosed early but its highly metastatic nature considerably worsens the prognosis [2]. Although several treatment regimens to target melanoma harboring the most prevalent BRAF mutation hold great promise with unprecedented response rates, treated patients ultimately develop resistance to therapy after a short period of disease control [3, 4]. However, intratumoral molecular heterogeneity in a BRAF-mutant melanoma implies a subpopulation of cells develop drug resistance, while another distinct population with different genetic component continues to grow and progress [5]. Therefore, identification and functional characterization of additional gene regulatory pathways to control melanoma growth and metastasis are essential to provide new therapeutic insights.

Our previous studies and others have demonstrated the crucial role of SOXE (Sry (Sex determining gene)-HMG box) E) members of the transcription factor family, SOX9 and SOX10, in NC development [6–8], which belongs to a transient and multipotent stem-like population that gives rise to the peripheral nervous system, craniofacial skeleton and melanocytes [9]. While SOX9 and SOX10 exhibit similar roles in NC specification and migration [6, 10, 11], the unique expression of SOX10 in embryonic and adult melanocytes dictates its functional requirement for their specification and homeostasis, respectively [12–15]. Consistently, previous studies demonstrated an essential role for SOX10 in the pathogenesis of melanoma in both mice and humans by promoting initiation, proliferation, survival, and invasion [16, 17]. Conversely, SOX9 is expressed in normal human melanocytes but its expression gradually downregulates as melanocytic cells progress from nevi to primary melanoma and are completely absent in the metastatic state, suggesting its negative role in melanoma progression. Indeed, overexpression of SOX9 in both human and mouse melanoma cell lines resulted in inhibition of cell proliferation and tumor growth in xenografts [18]. Another study showed that SOX9 and SOX10 play antagonistic functions in melanoma cells as demonstrated by upregulation of SOX9 expression, which contributed to the pro-apoptotic response induced by SOX10 loss-of-function. These findings indicate that SOX10 could promote melanoma initiation and progression by repressing SOX9 expression, which otherwise would have elicited anti-tumorigenic processes [17]. Moreover, previous studies identified that the melanoma inhibitory activity (MIA) protein was responsible for SOX10-mediated melanoma cell migration and invasion but ectopic expression of MIA could only partially restore the invasive capacity of SOX10-inhibited melanoma cells, suggesting the involvement of other SOX10 target genes [19].

On the contrary, other studies revealed that SOX9 was highly expressed in metastatic melanoma patient samples and contributed to melanoma invasion, suggesting that SOX9 is a negative prognostic factor in advanced melanoma [20, 21]. The discrepancies between the different studies could be attributed to melanoma heterogeneity with distinct expression levels of SOX9 and/or SOX10 in the tumors. Whether they share the same or different downstream targets in mediating melanoma growth and metastasis remain elusive.

Nedd9 (Neural precursor expressed, developmentally down-regulated 9), a member of the Crk-associated substrate (CAS) family of signal transduction proteins, has been demonstrated to function as a scaffolding protein to regulate NC migration and tumor progression in a variety of cancers including melanoma [22–28]. Our previous studies showed that SOX9 directly transactivates *NEDD9* expression to restrict polarized RHOA activity, which is essential for directional migration of mesenchymal NCCs [22]. Likewise, elevation of NEDD9 expression was detected in 30 to 50% of metastatic melanomas samples and promoted mesenchymal migration of melanoma cells through activation of RAC1 and inhibition of RHO/ROCK-driven amoeboid movement [29, 30]. Whether NEDD9 expression is also subjected to the transcriptional regulation by SOXE proteins in melanoma remain to be determined.

In this study, using antibodies specific for SOX9, SOX10, and NEDD9, we detected distinct but overlapping expression patterns of SOX10 and NEDD9 in nevi, primary and metastatic melanoma specimens, whereas SOX9 was predominantly and highly expressed in NEDD9^+^ metastatic melanoma in the small intestine and lung. Consistently, as demonstrated in the functional assays, we found NEDD9 expression is regulated by SOX10 and mediates its metastatic functions in melanoma cell lines. When *SOX10* expression was silenced, a moderate upregulation of *SOX9* expression level was observed and contributed to the anti-metastatic events. We revealed that further increased SOX9 dosage with comparable expression levels to a range of high *SOX9* mRNA detected in malignant melanoma specimens could restore the metastatic properties in *SOX10* knockdown cells, partly through induction of NEDD9 activity. Lastly, SOX10 or high SOX9 expression mediates melanoma cell migration through the NEDD9-regulated focal adhesion dynamics and Rho GTPase signaling. Taken together, these findings suggest that distinct levels of SOX9 expression determine whether it functions as a suppressor or an inducer of melanoma metastasis.

## Methods

### Melanoma specimens

Surgically procured tumor samples from patients with nevus, primary cutaneous and metastatic melanomas were obtained in the Department of Anesthesiology, Zhejiang Cancer Hospital and Department of Pediatric Surgery, the Second Hospital of Hebei Medical University with informed patients’ consent for research purposes. All biopsy samples were either fixed with formalin before embedding in the paraffin wax or processed for qPCR analysis.

### Constructs and cell lines

The human *SOX9* cDNA was cloned into the lentiviral pWPI vector (Addgene plasmid 12,254). The human *NEDD9* cDNA fragment was amplified using pEF-HEF1 as a template and cloned into lentiviral vector pLVX-EF1α-puro (Clontech). The shRNA against the human *SOX10* (5’-GACTTCGGCAACGTGGACATT-3′) and *NEDD9* (5’-GAGACACCATCTACCAAGTTT-3′) were designed based on the principles from The RNAi Consortium (https://www.broadinstitute.org/rnai/public/) and cloned into lentiviral vector pLKO.1-puro. pLKO.1-TRC control was gift from David Root (Addgene plasmid #10879).

Human epidermal melanocyte (HEMa-LP) was purchased from ThermoFisher and cultured in Medium-254 supplemented with HMGS-2. Human melanoma cell lines A375M, UACC-457, UACC-827, UACC-903 except SK-MEL-28 and human embryonic kidney cell line 293 T were cultured in DMEM medium with high glucose (Life Technologies) supplemented with 10% fetal bovine serum (FBS) (BioSera) and 100 U/ml penicillin-streptomycin (Life Technologies). RPMI-1640 medium (ThermoFisher) was used to culture Me300 kindly provided by D Leung, the Hong Kong University of Science and Technology and SK-MEL-28. Human melanoma cell line WM266–4 was obtained from ATCC and cultured in EMEM medium (Sigma) supplemented with 10% FBS and 100 U/ml penicillin-streptomycin. Cell lines were authenticated by cell profiling (AmpFISTR Identifier PCR Amplification kit, Life Technologies).

### Lentiviral transduction

For lentivirus production, 5 × 10^6^ 293 T cells were plated in a 100 mm dish and transfected with a lentiviral expression vector, packaging plasmid psPAX.2 and envelope plasmid pMD2.G using PolyJet™ (SignaGen). The cell culture medium containing the lentiviral particles was harvested 48 and 72 h post-transfection and filtered through a 0.22 μm filter. 3 × 10^5^ melanoma cells were infected with lentivirus particles expressing cDNA and/or shRNA and cultured in the presence of 8 μg/ml Polybrene (Sigma) for 24 h. After 48 h transduction, infected melanoma cells were screened in presence of 1 μg/ml puromycin (Life Technologies).

### Colony formation assay

Following puromycin selection of A375M and WM266–4 melanoma cells transduced with lentiviral particles expressing cDNA and/or shRNA, single cell suspension (5 × 10^2^) in complete medium (10% FBS in DMEM for A375M, 10% FBS in EMEM for WM266–4) was seeded in each well of a 6-well plate. Plates were incubated at 37 °C for 1 week for A375M and 2 weeks for WM266–4, during which culture medium was changed every 3 days. Following methanol (Merck) fixation and 0.1% crystal violet (Sigma) staining, the number of colonies formed in each well was calculated by Quantity One Software (Bio-Rad).

### AlamarBlue assay

Following puromycin selection of A375M and WM266–4 melanoma cells transduced with lentiviral particles expressing cDNA and/or shRNA, single cell suspension (1 × 10^3^) in complete medium (10% FBS in DMEM for A375M, 10% FBS in EMEM for WM266–4) was seeded in each well of a 96-well plate and incubated at 37 °C. After 24 h, each well was replaced with 100 μL of complete medium containing 10% AlamarBlue (Life Technologies) and incubated at 37 °C for 2 h. 10% AlamarBlue containing medium in each well was then transferred to a new 96-well plate for measurement of the absorbance reading at 570 nm and 600 nm. Cells were replaced with a fresh complete medium. 100 μL of 10% AlamarBlue containing medium was added to each well and measured 2 h post incubation every 24 h for 4 to 9 days in order to determine the growth curve of the cells with different treatments based on the AlamarBlue absorbance rate between 570 and 600 nm according to manufacturer’s instruction.

### Transwell invasion assay

Following puromycin selection of A375M and WM266–4 melanoma cells transduced with lentiviral particles expressing cDNA and/or shRNA, single cell suspension (5 × 10^4^) in plain medium (DMEM for A375M, EMEM for WM266–4) was seeded on the transparent PET membrane of cell culture insert (8 μm, Falcon). For the invasion assay, the membrane was coated with 150uL of Matrigel (2.5 mg/mL, Corning) on the ice and gelling at 37 °C for 6 h prior to seeding. Cells were allowed to invade through the membrane driven by FBS in the lower chamber for 12 h (A375M) or 48 h (WM266–4). Cells failed to invade were removed by the cotton swap. After 100% methanol fixation and DAPI (1 μg/ml, Sigma) staining, the number of invaded cells was counted in 15 random fields within the membrane under an inverted fluorescence microscope.

### Western blot

Cells were washed twice with cold phosphate-buffered saline (PBS) and lysed in RIPA buffer (150 mM NaCl, 1 mM EDTA, 1% NP40, 0.5% Sodium deoxycholate, 0.1% SDS, 50 mM Tris-HCl, pH 7.5) supplemented with 1% protease and phosphatase inhibitor cocktail (ThermoFisher). Proteins were separated by SDS-PAGE using the Bio-Rad system under reducing conditions. Membranes were probed with antibodies against SOX9 (H-90, Santa Cruz), SOX10 (N-20, Santa Cruz), NEDD9 (Clone 2G9, Abcam) and GAPDH (FL-335, Santa Cruz) for overnight at 4 °C and then incubated with appropriate horseradish peroxidase-conjugated goat anti-rabbit, rabbit anti-mouse and donkey anti-goat antibodies (at 1:2000, Dako) at room temperature for 1 h. After incubation with ECL substrate (WesternBright, Advansta) for 1–3 min, blots were exposed to X-ray film (FujiFilm Super RX) at different times to obtain the optimal intensity of the protein bands which were analyzed by ImageJ.

### Quantitative polymerase chain reaction (qPCR)

Total RNA was extracted using MiniBEST Universal RNA Extraction Kit (Takara) and reverse transcribed for cDNA synthesis using PrimeScript RT Master Mix (Takara). All reactions including non-template controls were performed in triplicate on StepOnePlus Real-time PCR system (Applied Biosystem) using SYBR Premix Ex Taq II (Takara). Human 36B4 was used for normalization. List of primers for detection of gene expression is listed below.

**Table:** 

Gene	Species	Probe length (bp)	Primers (5′-3′)
*SOX10*	Human	83	For: GACCAGTACCCGCACCTG
			Rev: CGCTTGTCACTTTCGTTCAG
*SOX9*	Human	102	For: ACACACAGCTCACTCGACCTTG
			Rev: GGAATTCTGGTTGGTCCTCTCTT
*NEDD9*	Human	159	For: ATGTCCACGTCTTCCACCTCC
			Rev: AGTGACCAGTGCCATTAGGCTG
*36B4*	Human	101	For: GTGATGTGCAGCTGATCAAGACT
			Rev: GAAGACCAGCCCAAAGGAGA
*MMP1*	Human	111	For: AGGTCTCTGAGGGTCAAGCA
			Rev: CTGGTTGAAAAGCATGAGCA
*MMP2*	Human	148	For: AAGAAGTAGCTGTGACCGCC
			Rev: TTGCTGGAGACAAATTCTGG
*MMP3*	Human	138	For: ATTCCATGGAGCCAGGCTTTC
			Rev: CATTTGGGTCAAACTCCAACTGTG
*MMP7*	Human	158	For: GAGTGAGCTACAGTGGGAACA
			Rev: CTATGACGCGGGAGTTTAACAT
*MMP8*	Human	154	For: TCTGCAAGGTTATCCCAAGG
			Rev: ACCTGGCTCCATGAATTGTC
*MMP23*	Human	144	For: CCAGAAGATCCTCCACAAGA
			Rev: CAGGTGTAGGTGCCCTCATT

### Luciferase reporter assay

A375M and WM266–4 melanoma cells were transfected with *FireFly* luciferase reporter vector driven by human *NEDD9* proximal promoter (~ 1 kb) and *Renilla* luciferase reporter vector using PolyJet transfection reagent based on manufacturer’s protocol. Cells were harvested and lysed 48 h post-transfection. The cell lysate was measured by PerkinElmer Victor 3 Multi-label Plate Reader using Dual-Luciferase Reporter Assay System (Promega) according to the manufacturer’s instructions. The luminescence signal of the *Renilla* luciferase reporter activity was used for normalization of *FireFly* luciferase reporter activity.

### Chromatin immunoprecipitation

A375M melanoma cells were transduced with SOX10 or SOX9-overexpressing lentivirus. A total of 6 × 10^6^ cells for each treatment were fixed by 1% formaldehyde and lysed, then digested using micrococcal nuclease according to the manufacturer’s protocol (Pierce Agarose ChIP Kit, 26,156, Thermofisher). Supernatant was collected and sonicated for 6 × 30 s in a Bioruptor sonicator (Diagenode). The target size of chromatin fragments ranging from 400 bp to 600 bp was confirmed by 2% agarose gel electrophoresis. Chromatin fragments were immunoprecipitated by using normal rabbit IgG control (Thermofisher), 2 μg anti-SOX10 antibody (ChIP grade, sc-17,342X, Santa Cruz) or 2 μg anti-SOX9 antibody (ChIP grade, AB5535, Millipore) at 4 °C overnight. 20uL of ChIP grade Protein A/G Plus Agarose (Thermofisher) was added into the chromatin-antibody mixture and incubated at 4 °C for 2 h. DNA fragments were then purified and recovered based on manufacturer’s instruction, followed by 40 cycles of quantitative PCR. Primers used for amplification of fragments covering SOX binding motif (AAACAAA) are: 5′- GGAGGGCCACTAGCTAGAGA-3′ and 5’-GCCTCCAAGAGATCTAGATAAC-3′. Primers targeting non-SOX binding motif are 5′- TTTTCGCCTCACTGCTCTGT-3′ and 5′- GGCTGGCATTTCTAGCTCCA-3′. Data were analyzed and presented as the fold enrichment relative to IgG control.

### Time-lapse imaging of melanoma cell migration in a wound-healing assay

A375 melanoma cells transduced with Lifeact-mCherry together with different constructs were cultured in a 10cm^2^ dish until 90% confluent. A migration gap of approximately 1 mm was then created by introducing a “scratch” to the adherent layer of cultured cells using a sterile 200 μL pipette tip. At this point, half of the culture medium was removed and replaced with fresh medium to reduce the number of cells introduced into suspension reattaching to the cell-free zone during experimentation. Time-lapse imaging of melanoma cells migrating to the wound was performed on a Perkin Elmer Widefield imaging microscope equipped with an incubator capable of maintaining 37 °C temperature, 95% relative humidity and 5% CO2. Images were acquired with 10X objective and collected every 5 min for a total period of 7.5 h at one time. Images were analyzed by Image J software. The cell speed was determined by the total distance traveled divided by the time.

### RHOA and RAC1-activation assay

Detection of RHOA and RAC1 activity was performed by RHOA and RAC1 Pull-down Activation Assay Biochem Kits (Cytoskeleton) based on manufacturer’s protocol. Melanoma cells at 80% confluence were washed twice with cold PBS and lysed with cold Cell Lysis Buffer supplied with 1% Protease and Phosphatase Inhibitor Cocktail for 3 min on ice. Cell lysates were then centrifuged at 10,000 g for 3 min. The supernatant (600 μg) was then immediately incubated with Rhotekin-RBD (50 μg) or PAK-PBD (20 μg) beads at 4 °C overnight. Active RHOA and RAC1 were pull downed by the Rhotekin-RBD and PAK-PBD beads respectively, which were washed six times by wash buffer followed by western blotting using mouse monoclonal antibodies against RHOA (Santa Cruz) and RAC1 (Santa Cruz).

### Immunofluorescence

After deparaffinization and rehydration, the paraffin melanoma sections (5 μm) were subjected to antigen retrieval by boiling in Target Retrieval Solution (Citrate pH 6.0, Dako) for 10 min and cooled down to room temperature for 30 min. Sections were blocked in 1% normal donkey serum with 0.1% TritonX-100 and followed by 4 °C overnight incubation of primary antibodies (diluted in 1% normal donkey serum PBS) against SOX9 (1:100, H-90, Santa Cruz), SOX10 (1:200, N-20, Santa Cruz) and NEDD9 (1:1000, Clone 2G9, Abcam). Anti-rabbit, anti-mouse and anti-goat antibodies conjugated with Alexa-488, Alexa-555 and Alexa-647 were applied as secondary antibodies. For staining of focal adhesion and stress fiber, 1 × 10^4^ melanoma cells were seeded onto sterile coverslips in 24-well plate 24 h prior to 4% formaldehyde fixation on ice for 30 min. Cells on coverslip were blocked by 1% bovine serum albumin with 0.03% TritonX-100 in PBS, followed by 4 °C overnight incubation of anti-Vinculin (1:500, Invitrogen) and then one-hour room temperature incubation of Alexa Fluor 546 Phalloidin (1:500, Cytoskeleton). DAPI (1 μg/ml, Sigma) was used as a nuclear counter-stain for both immunostained sections and cells on coverslips. Fluorescence images were captured using Carl Zeiss LSM 780 confocal microscope and analyzed by ZEN 2011 and MetaMorph software in the Faculty Core Facility, Li Ka Shing Faculty of Medicine, University of Hong Kong.

### In vivo pulmonary metastasis assay

The following mouse experimentation was approved by the Committee on the Use of Live Animals in Teaching and Research (CULATR), University of Hong Kong (CULATR no: 4005–16).

After lentiviral transduction and puromycin selection, a 100 μL single cell suspension containing 1 × 10^6^ A375M cells in plain DMEM was injected into the tail vein of 7-week-old female NOD-SCID mice. Four weeks post-injection, mice were anesthetized before intraperitoneal injection of 100uL of sterile D-Luciferin firefly potassium salt solution (30 mg/mL). The tumor cells colonized in the lung as reflected by bioluminescent signals were acquired for 4 min in vivo imaging using Xenogen IVIS 200. Regions of interest (ROI) were manually selected, and the results were quantified as the average radiance of photons emitted per second and area by using the Living Image software (Xenogen, Alameda, CA). After quantification, mice were sacrificed and the lung nodules were counted.

### Statistical analysis

The experiments were performed at least three times independently. The statistical data were expressed as Mean ± SD (SD = standard deviation of the mean values of each independent experiments). Student’s t-test and two-way analysis of variance (ANOVA) were used to determine the confidence levels for group comparison. Values are statistically significant at p* < 0.05; **p < 0.01; ***p < 0.001.

## Results

### Positive correlation of expression between SOXE and NEDD9 in melanoma patient specimens

**Fig. 1:**
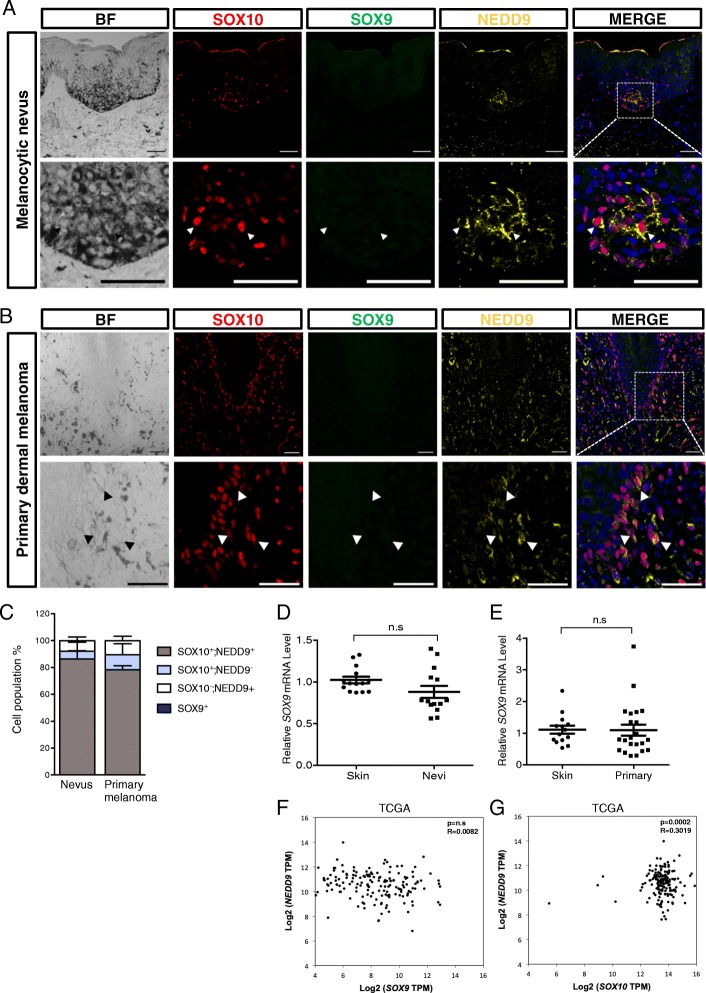
Co-expression of SOX10 and NEDD9 but not SOX9 in melanocytic nevi and primary dermal melanomas. **a**, **b** Representative images showing immunofluorescence for SOX10, SOX9, and NEDD9 in the skin sections of patients with benign melanocytic nevus (**a**) and primary dermal melanoma (**b**). White arrowheads indicate cells coexpressing SOX10 and NEDD9 but not SOX9. The dotted white box in the merged image indicates the magnified region with separate color channels shown in the lower panels. Cell nuclei were counterstained by DAPI (blue). Scale bars: 10 μm. **c** Quantification of the number of cells positive for the indicated markers in 12 melanocytic nevi and 14 primary dermal melanoma samples. **d**, **e** qPCR analysis of *SOX9* expression in 14 healthy skin controls, 14 melanocytic nevi, and 22 primary melanoma samples. **f** Correlation expression analysis between *SOX9* and *NEDD9*; *SOX10* and *NEDD9* (**g**) in melanoma patient samples obtained from Skin Cutaneous Melanoma dataset in TCGA (173 patients). Error bars represent mean ± SD. n.s, non-significant. The *P*-value and Pearson correlation coefficient are denoted on top

**Fig. 2:**
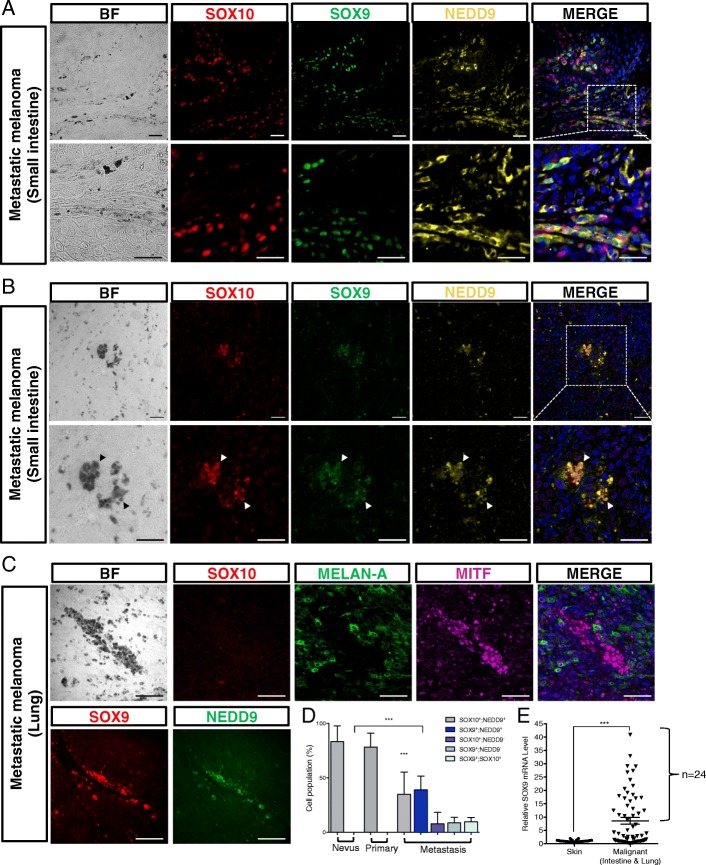
SOX9 expression in metastatic melanomas. **a**, **b** Representative images showing immunofluorescence for SOX9, SOX10, and NEDD9 in the cross-section of the intestinal metastatic melanoma from two patients. **b** Arrowheads indicate pigmented melanoma cells co-expressing SOX9, SOX10, and NEDD9. The dotted white box in the merged image indicates the magnified region with separate color channels shown in the lower panels. **c** Representative images showing immunofluorescence on consecutive lung sections with metastatic melanoma for SOX10, MELAN-A, MITF, as well as SOX9 and NEDD9. Cell nuclei were counterstained with DAPI (blue). Scale bars: 10 μm. **d** Quantification of the number of cells positive for the indicated markers in 12 melanocytic nevi, 14 primary dermal melanomas and 25 metastatic melanomas. **e** The amount of *SOX9* transcripts was measured by qRT-PCR in 22 healthy skin controls, metastatic melanoma specimens from the intestine (*n* = 37) and lung (*n* = 27). Error bars represent mean ± SD. Student‘s *t-test*, ***p < 0.001

Our recent studies demonstrated that *NEDD9* is a direct transcriptional target of SOX9 in mediating neural crest delamination [22]. In addition, NEDD9, SOX9, and SOX10 have been shown to be crucial for human melanoma metastasis [20, 28]. Whether SOX9 and/or SOX10 exhibit a similar regulatory relationship with NEDD9 in melanoma has not yet been examined. To address this issue, we first performed comparative expression study of these factors on tissue sections from Chinese patients with benign melanocytic nevus, primary dermal and metastatic melanomas using antibodies specific for SOX9, SOX10, and NEDD9 [17, 31]. Immunofluorescence staining showed that NEDD9 was localized in the cytoplasm and co-expressed with most, if not all, of SOX10^+^ pigmented nevus and primary melanomas, whereas SOX9 was barely detectable (Fig. 1a-c). In agreement with this, we performed qPCR for *SOX9* on a cohort of melanocytic nevi and primary melanomas and the majority of which remained at the basal level of expression (Fig. 1d, e). Similarly, analyzing the Cancer Genome Atlas (TCGA) dataset from 173 patients with primary cutaneous melanomas revealed no significant correlation of expression between *SOX9* and *NEDD9* whereas we observed a moderate but significant correlation of expression between *SOX10* and *NEDD9* (Fig. 1f, g). In contrast, SOX9 expression began to express in a subset of NEDD9^+^ melanoma cells, which had metastasized to the small intestine and another subset of NEDD9^+^ cells exhibited SOX10 expression (Fig. 2a, d), whereas we detected co-expression of SOX10, SOX9, and NEDD9 in another patient with intestinal melanomas (Fig. 2b, d). In another sample, we found no SOX10 expression in lung metastases where subset of pigmented cells exhibited co-expression of SOX9 and NEDD9 (Fig. 2c, d). Although these cells were negative for a melanoma marker MELAN-A [32], they were positive with a diagnostic marker for metastatic melanoma MITF [33] (Fig. 2c). Moreover, there was no strict correlation between the patterns of SOX9, SOX10, and NEDD9 expression and distribution of pigmented melanomas in all examined stages (Fig. 1a, b, and Fig. 2a-c). Taken together, our immunofluorescence studies demonstrate a positive correlation between NEDD9 and SOX10 expression in human tissue biopsies from different stages of melanoma progression including common acquired nevi, primary melanoma, and metastases, while SOX9 expression appears to associate with a more invasive and metastatic phenotype. Indeed, qPCR analysis revealed that elevated SOX9 expression was frequently detected in lung and intestine metastatic melanomas compared to the control skin samples from healthy subjects (Fig. 2e). These results demonstrate that high SOX9 expression is predominantly associated with metastatic melanomas.

### Upregulated level of *SOX9* expression contributes to cell growth arrest, reduced migratory capacity and colony formation activity in *SOX10* knockdown melanoma cells

**Fig. 3:**
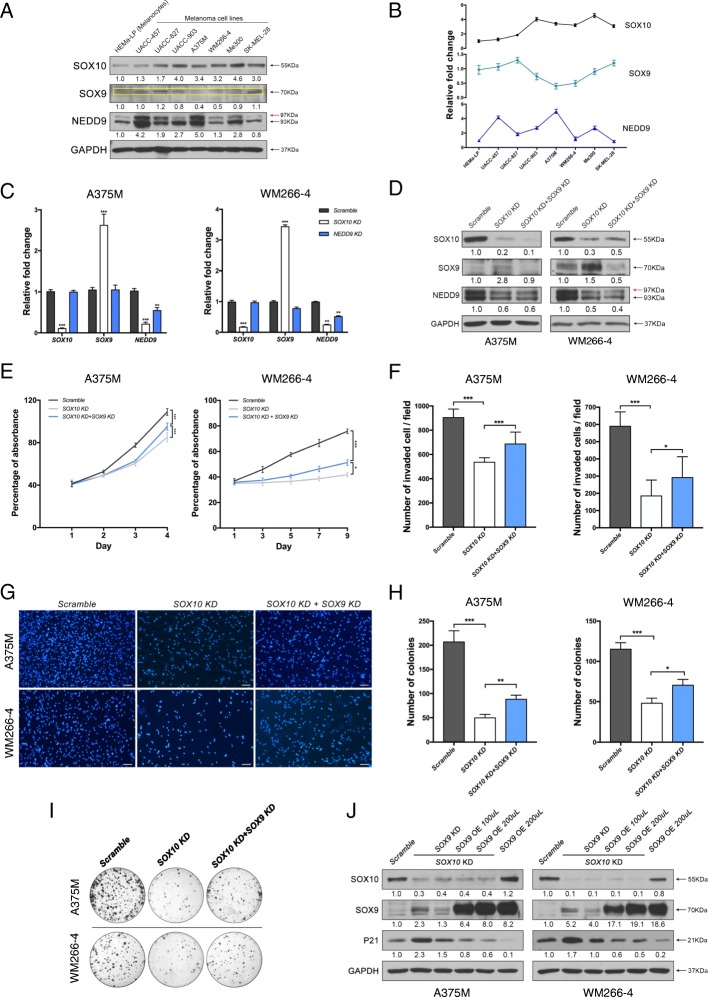
Upregulated or low level of *SOX9* expression contributes to the anti-metastatic/anti-oncogenic activities of *SOX10* knockdown (KD) melanoma cells. **a** Expression of SOX10, SOX9, and NEDD9 in human melanocytes (HEMa-LP), and a panel of metastatic melanoma cell lines. GAPDH was used as a loading control. The yellow box indicates protein bands corresponding to the size of SOX9. The red arrow indicates a phosphorylated form of NEDD9. The intensity of protein bands in arbitrary units for SOX10, SOX9, and NEDD9 in each melanoma cell line is relative to HEMa-LP which is set to 1 as a reference. **b** Line plots represent the intensity of protein bands shown in (**a**). **c** qRT-PCR analysis of *SOX10*, *SOX9* and *NEDD9* transcript levels in A375M and WM266–4 cells treated with scramble control, *SOX10* KD and *NEDD9* KD. Data represent fold change normalized to scramble control and the average of three independent assays. **d** Western blot analysis of SOX9, SOX10 and NEDD9 protein levels in each cell line transduced with scramble control, *SOX10* KD and *SOX10* KD + *SOX9* KD. GAPDH serves as a loading control. The red arrow indicates a phosphorylated form of NEDD9. AlamarBlue (**e**), transwell invasion (**f**, **g**) and colony formation assays (**h**, **i**) of each cell line treated with scramble control, *SOX10* KD and *SOX10* KD + *SOX9* KD. **g** DAPI images of transwell invasion of melanoma cells treated with the indicated constructs. Scale bars: 100 μM (**i**) Representative images showing crystal violet stained colonies formed from A375M and WM266–4 cells treated with scramble control, *SOX10* KD and *SOX10* KD + *SOX9* KD. **j** Western blot analysis of SOX10, SOX9 and p21 protein levels in each cell line transduced with scramble control, *SOX10* KD alone, *SOX10* KD + *SOX9* KD, two different volume (100 μL, 200 μL) of lentiviruses encoding *SOX9* gene (*SOX9* OE) in *SOX10* KD and maximum dose of *SOX9* OE in parental cells. GAPDH serves as a loading control. Error bars represent mean ± SD of three independent experiments. *p < 0.05, **p < 0.01, ***p < 0.001

The predominant association of SOX10 and NEDD9 but not SOX9 expression in melanoma specimens is further supported by co-expression of these two factors at different levels in a series of malignant melanoma cell lines (Fig. 3a, b). SOX10 levels were higher in all melanoma cell lines than in normal human melanocytes (HEMa-LP), whereas SOX9 expression was low in all these cell lines. These prompted us to examine whether SOX10 regulates NEDD9 expression in two metastatic melanoma cell lines (A375M and WM266–4) harboring mutated BRAF, which express high levels of SOX10 and NEDD9 expression (Fig. 3a, b). We first analyzed *NEDD9* expression in these cell lines transduced with lentiviral-shRNA-scramble control or shRNA-mediated knockdown of *SOX10* (*SOX10* KD). We observed a significant reduction of *NEDD9* transcripts in *SOX10* KD compared to control, suggesting that *NEDD9* expression could be regulated by SOX10 in melanoma cells. In contrast, *SOX9* expression was significantly upregulated in *SOX10* KD (Fig. 3c), consistent with previous observations that SOX10 normally suppressed SOX9 expression which otherwise would have elicited a pro-apoptotic response in melanoma cells [17]. Importantly, the upregulated levels of *SOX9* expression in *SOX10* KD A375 (1.5 to 2.6 fold) and WM266–4 (1.5 to 3.4 fold) are clinical relevant as they fall within the range of *SOX9* expression levels detected in some specimens of primary melanoma (1.3 to 3.7 fold) (Fig. 1e and Fig. 3c). There was no significant difference in the degree of reduced NEDD9 expression between *SOX10* KD and *SOX9* KD + *SOX10* KD, suggesting that upregulated level of SOX9 expression did not contribute to the reduction of NEDD9 expression in *SOX10* KD cells (Fig. 3d). To further expand the studies on the anti-tumorigenic effects of increased *SOX9* levels in *SOX10* KD, we performed *SOX9* KD in both *SOX10* KD A375M and WM266–4 melanoma cells and compared the effects with *SOX10* KD alone and control on cell proliferation, invasion and oncogenicity using alamarBlue, transwell and colony formation assays respectively. While *SOX10* KD resulted in a marked reduction of cell growth (Fig. 3e), invasive capacity (Fig. 3f, g), and colony formation (Fig. 3h,i), *SOX9* KD partly restored these properties in *SOX10* KD cells (Fig. 3e-i). These results further confirm that the upregulated *SOX9* expression contributes to the anti-tumorigenic and anti-metastatic effects of *SOX10* KD melanoma cells.

On the other hand, previous studies showed that either overexpression of SOX9 alone or upregulation of *SOX9* expression in *SOX10* KD caused cell cycle arrest through an increase in cyclin-dependent kinase inhibitor p21 protein expression in melanoma cell lines [16, 18]. These results prompted us to further examine whether the levels of SOX9 expression determine the degree of p21 induction. In agreement with previous observations, we observed a pronounced elevation of p21 protein upon *SOX10* KD in both A375M and WM266–4 melanoma cells compared to the scramble control, whereas increased p21 expression was alleviated by *SOX9* KD (Fig. 3j), indicating that upregulation of endogenous *SOX9* by *SOX10* KD is required for the induction of p21 expression. We then further applied the amount of lentiviral vector expressing SOX9 (*SOX9* OE) at two different titers (100 μL and 200 μL) in the *SOX10* KD cells, and that resulted in increased *SOX9* mRNA levels by 12 and 33-fold in A375, and 13 to 44-fold in WM266–4 respectively (Fig. 5a). These levels fall within the range of high *SOX9* expression being detected in the metastatic melanoma samples (10 to 44-fold vs normal skin, Fig. 2e). The results showed that the progressive elevation of SOX9 expression levels caused a gradual reduction of p21 expression (Fig. 3j). Moreover, we obtained a greater degree of reduction in p21 expression level at the highest dose (200 μL) of *SOX9* OE in parental cells (Fig. 3j). Altogether, these data demonstrate that in the absence of *SOX10*, the upregulated or low levels of endogenous *SOX9* expression promote p21 expression whereas high levels of SOX9 could inhibit p21 expression and may enhance cell growth.

### NEDD9 functions downstream of SOX10

**Fig. 4:**
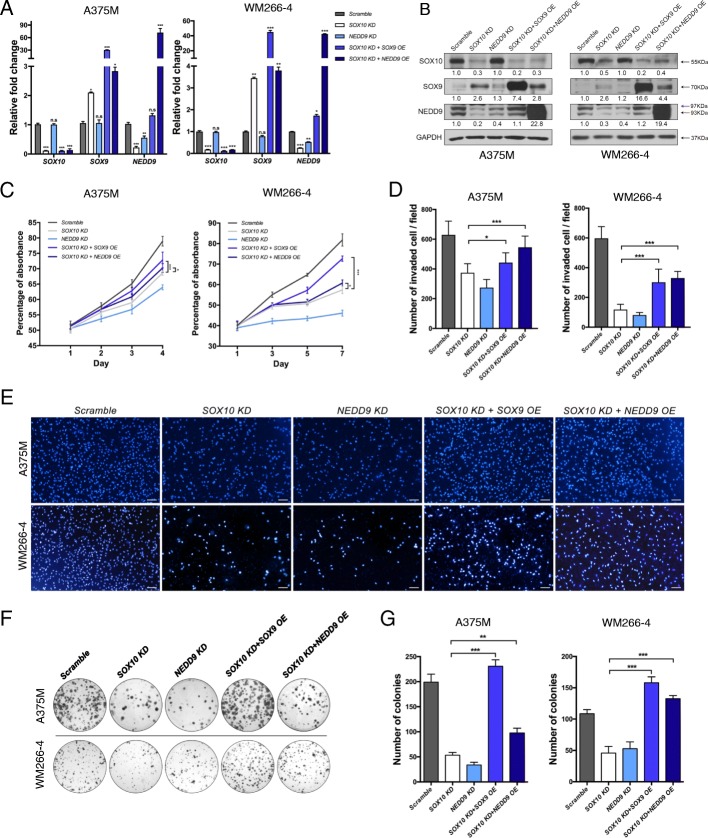
Overexpression of SOX9 and NEDD9 restore the oncogenic features of *SOX10* KD melanoma cells. qRT-PCR (**a**) and Western blot (**b**) analysis for the expression levels of SOX10, SOX9 and NEDD9 in A375M and WM266–4 cell lines treated with the indicated constructs. Data are fold change normalized to scramble control and the average of three independent assays. The red arrow indicates the phosphorylated form of NEDD9. GAPDH serves as a loading control. AlamarBlue (**c**) and transwell invasion assays (**d**) of each cell line treated with the indicated constructs. **e** DAPI images of transwell invasion of melanoma cells treated with the indicated constructs. Scale bars: 100 μM. **f** Representative images of crystal violet stained A375M and WM266–4 clones subjected to different treatments. **g** Quantification of the number of A375M and WM266–4 colonies treated with the indicated constructs. Error bars represent mean ± SD of three independent experiments. *p < 0.05, **p < 0.01, ***p < 0.001

The downregulation of *NEDD9* expression in *SOX10* KD prompted us to examine whether *NEDD9* knockdown (*NEDD9* KD) would exhibit a similar functional outcome as in *SOX10* KD. qPCR and Western blot analysis of A375M and WM266–4 cells treated with shRNA-*NEDD9* showed a significant reduction in the levels of NEDD9 RNA and protein of both parental and phosphorylated forms compared with the scramble control respectively (Fig. 4a,b). In vitro functional studies further showed that *NEDD9* KD caused a marked reduction of cell growth (Fig. 4c), invasive behavior (Fig. 4d,e) and colony formation capacity (Fig. 4f,g) as observed in *SOX10* KD (Fig. 4c-g), suggesting that NEDD9 is required for proliferation, invasion and oncogenicity of melanoma cells. Importantly, *NEDD9* KD did not significantly alter the transcript and protein levels of SOX9 and SOX10 (Fig. 4a,b). These results suggest that NEDD9 could function downstream of SOX10 to mediate its tumorigenic effects. Indeed, overexpression of *NEDD9* gene (*NEDD9* OE) restored cell growth, invasive capacity and colony formation activity in *SOX10* KD cells to a different extent depending on cell lines (Fig. 4c-g). NEDD9 OE exhibited a similar degree of rescue growth and invasive capacity in both A375M and WM266–4 cells (Fig. 4c-e). In contrast, only WM266–4 showed a complete restoration in the number of colonies formed in *SOX10* KD by NEDD9 OE while the only partial rescue was observed in A375M cells (Fig. 4f,g). Importantly, the restoration of oncogenic phenotypes in *SOX10* KD + NEDD9 OE cells was not due to an increase in *SOX9* expression level which is comparable to that in *SOX10* KD cells (Fig. 4a,b). While *SOX9* KD partly alleviated the anti-tumorigenic effects of *SOX10* KD without altering NEDD9 expression (Fig. 3d), NEDD9 OE was able to further restore the proliferation, invasive and colony formation capacity in *SOX10* KD + *SOX9* KD cells (Additional file 1). Altogether, these results indicate that NEDD9 can mediate most if not all of the metastatic and tumorigenic functions of SOX10.

### High level of SOX9 expression is metastatic and oncogenic

Although our previous studies in chick embryos demonstrated that overexpression of SOX9 was sufficient to induce ectopic *Nedd9* expression [22], our data showed that moderate increase of *SOX9* expression in *SOX10* KD melanoma cells was not able to restore *NEDD9* expression (Fig. 3c,d and Fig. 4a,b), raising the possibility that further elevation of SOX9 expression level might be required for the restoration of NEDD9 expression based on the previous findings that SOX9 acts in a dose-dependent manner [34, 35]. Indeed, *SOX9* OE at the highest titer (200 μL) in both *SOX10* KD A375M and WM266–4 melanoma cells significantly restored the levels of NEDD9 mRNA and protein expression of both parental and phosphorylated forms compared to *SOX10* KD alone (Fig. 4a,b). Consistently, *SOX9* OE restored proliferation and colony formation of *SOX10* KD cells to a greater extent (Fig. 4c,f,g), but partly rescued on invasion in both *SOX10* KD A375M and WM266–4 cells (Fig. 4d,e). These in vitro findings suggest that high level of SOX9 expression is metastatic/tumorigenic in melanoma cells.

### SOX9 transactivates NEDD9 expression in a dose-dependent manner

**Fig. 5:**
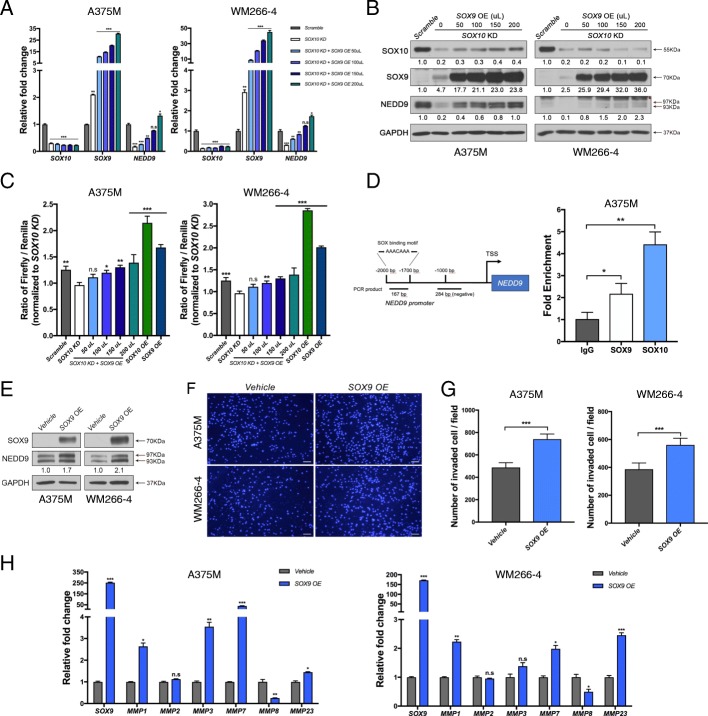
Overexpression of SOX9 transactivates *NEDD9* and induces *MMPs* expression. **a** qPCR analysis for the expression levels of *SOX10*, *SOX9*, and *NEDD9* in A375M and WM266–4 cell lines treated with scramble control, *SOX10* KD alone and *SOX10* KD together with increasing amount of *SOX9* OE lentiviruses. **b** Immunoblotting for the indicated antibodies on protein lysates derived from A375M and WM266–4 cells treated with the indicated constructs. The intensity of protein bands in arbitrary units for SOX10, SOX9, and NEDD9 in each melanoma cell line is relative to scramble control which is set to 1 as a reference. The red arrow indicates the phosphorylated form of NEDD9. Asterisk indicates non-specific bands. GAPDH serves as a loading control. **c** A375M and WM266–4 cells were transfected with a *1 kb-NEDD9* promoter-driven luciferase reporter construct plus renilla for normalization of transfection efficiency together with scramble control, *SOX10* KD, *SOX10* KD plus increasing amount of SOX9 OE lentiviruses, SOX10 OE, and SOX9 OE. Fold activation of three independent luciferase assays. *SOX10* KD is set to 1 as a reference. **d** Schematic diagram showing the presence of a SOX binding motif within the 167 bp DNA fragment detected by ChIP-qPCR whereas the 284 bp fragment serves as a negative control. ChIP-qPCR data showing a higher DNA binding capacity by SOX10 than SOX9. Anti-IgG serves as a negative control. **e** Western blot analysis using the indicated antibodies on protein lysates derived from A375M and WM266–4 cells treated with vehicle control and SOX9 OE. The red arrow indicates the phosphorylated form of NEDD9. **f** DAPI images of transwell invasion of melanoma cells treated with the indicated constructs. **g** Transwell invasion assay for each cell line treated with the vehicle alone and *SOX9* OE lentivirus. Scale bars: 100 μM. **h** mRNA expression of *SOX9* and members of MMP family were quantified by qRT-PCR in A375M and WM266–4 cells treated with the vehicle alone and *SOX9* OE. Error bars represent mean ± SD of three independent experiments. n.s, non-significant; *p < 0.05, **p < 0.01, ***p < 0.001

To further demonstrate the dose-dependent effects of SOX9 on NEDD9 expression, we transduced *SOX10* KD A375M and WM266–4 melanoma cells with a gradual increase in titer of lentiviral vector expressing SOX9 (50 μL to 200 μL) followed by assessment of *SOX9*, *SOX10* and *NEDD9* genes and protein expression levels (Fig. 5a,b). The results showed that progressive increase in the amount of lentiviruses expressing SOX9 resulted in a dose-dependent increase in the expression levels of SOX9 and NEDD9 in the SOX10 low environment (Fig. 5a,b), indicating that *SOX9* OE is able to restore NEDD9 expression in a dose-dependent manner in *SOX10* KD melanoma cells.

To further determine whether SOX10 and/or SOX9 can regulate NEDD9 expression through transactivating its promoter, we performed luciferase reporter assay driven by the NEDD9 promoter (~ 1 kb) in both A375M and WM266–4 melanoma cell lines. The results showed that *SOX10* OE (200 μL) exhibited a greater extent than *SOX9* OE (200 μL) in the transactivation of the *NEDD9* promoter activity in parental cells (Fig. 5c). In agreement with this, chromatin immunoprecipitation (ChIP) assay in A375M cells indicated that SOX10 has a higher binding affinity than SOX9 for a SOX consensus motif (AAACAAA) within the *NEDD9* promoter compared to IgG control (Fig. 5d), whereas none of these proteins bound to another DNA fragment without the motif, indicating the specificity of the binding (data not shown). In contrast, *SOX10* KD significantly reduced the *NEDD9*-reporter activity compared to the control, while *SOX9* OE restored the reporter activity in a dose-dependent manner in both cell lines (Fig. 5c). These data further confirm that high levels of SOX9 expression were able to induce and restore NEDD9 expression partly through binding and transactivating its promoter in both wild-type and SOX10 low environment, respectively (Fig. 5a-e).

Consistent with the observations that *SOX9* OE was able to restore the invasive behavior of *SOX10* KD A375M and WM266–4 cells, *SOX9* OE was also sufficient to promote the invasiveness of their parental forms compared to vehicle control (Fig. 5f,g). We then examined the impact of *SOX9* OE on a panel of matrix metalloproteinases (MMPs) expression which has been implicated in promoting melanoma metastasis through proteolysis of extracellular matrix [36]. qPCR analysis revealed that *MMP1*, *MMP7*, and *MMP23* expression were upregulated in both cell lines treated with *SOX9* OE compared to the vehicle control, whereas *MMP8* expression was downregulated. However, only A375 but not WM266–4 cells exhibited a robust elevation of *MMP3* expression in response to SOX9 OE (Fig. 5h). These findings are consistent with the roles of MMP1, MMP3, and MMP7 as pro-metastatic factors [37–39], and MMP8 as a negative regulator in melanoma invasiveness [40]. Intriguingly, the high MMP23 expression is associated with poor responses to immunotherapy [41]. Altogether, these results suggest that SOX9 OE not only promotes melanoma invasion through modulation of various *MMP* genes expression but also may have a role in immunosuppression.

### SOX9 overexpression restores metastasis in *SOX10* silenced melanoma cells in vivo

**Fig. 6:**
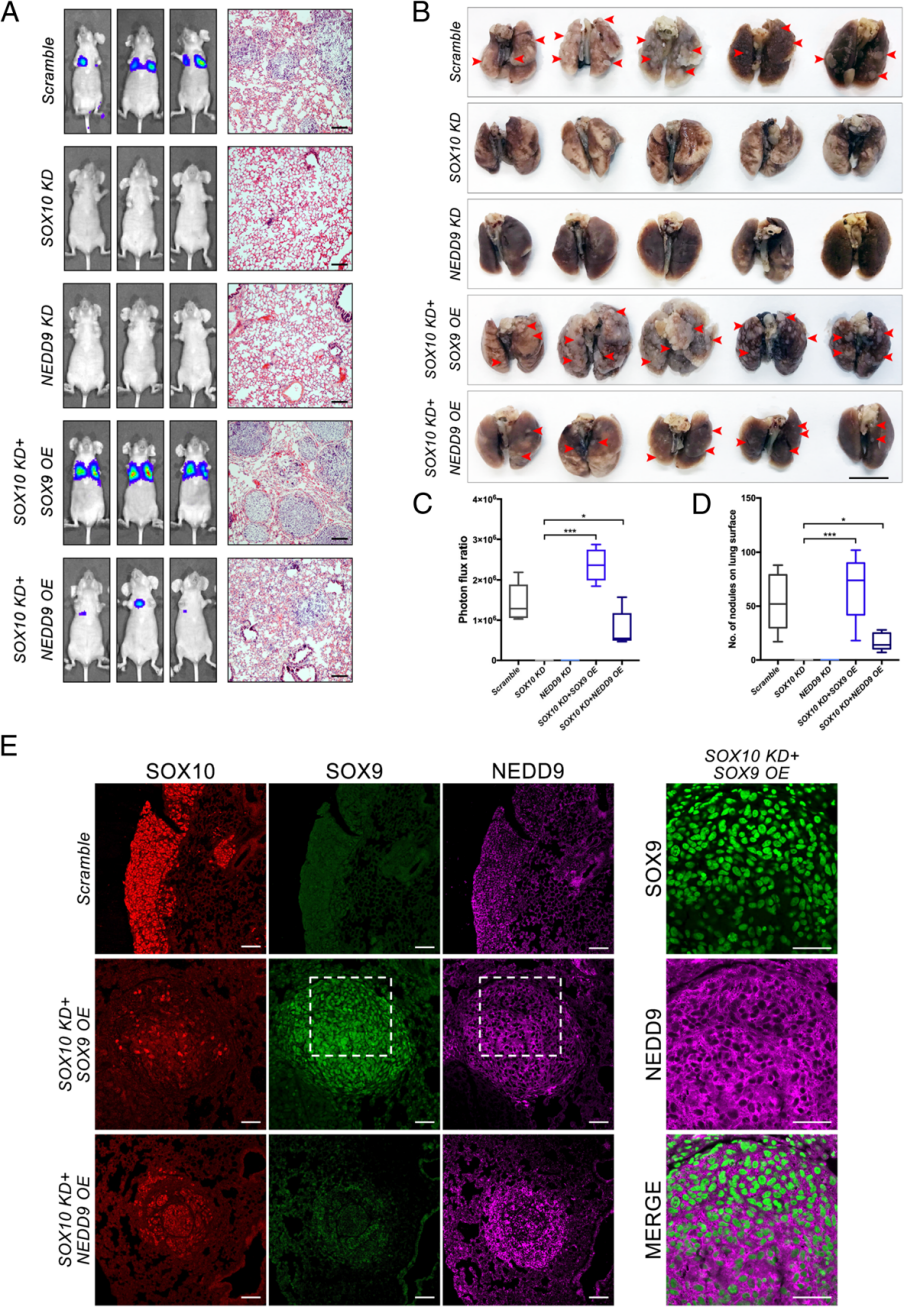
Overexpression of SOX9 restores lung metastasis in *SOX10* KD melanoma cells. **a** A375M cells treated with the indicated constructs were injected via the tail vein into NOD/SCID mice (*n* = 5 per treatment) and the bioluminescence images were taken 2 weeks after injection. H&E staining was used to detect for the presence of tumor tissues in lungs of NOD/SCID mice. Scale bars: 200 μM. **b** Gross pictures of lungs from xenografted NOD/SCID mice. The red arrowheads indicate the macroscopic lesions. Scale bar: 1.0 cm. **c** Bioluminescence intensity was measured and plotted. **d** Quantification of the number of nodules formed on the lung surface of NOD/SCID mice injected with the indicated constructs. **e** Immunofluorescence detection for SOX10, SOX9, and NEDD9 on the cross-section of lung nodules derived from A375M cells treated with scramble control, *SOX10* KD + *SOX9* OE and *SOX10* KD + *NEDD9* OE. Magnification of the boxed regions with an overlapping expression of SOX9 and NEDD9. Scale bar: 100 μM. Error bars represent mean ± SD of three independent experiments. *p < 0.05, p*** < 0.001

The restoration of metastatic capacity in *SOX10* KD cells in vitro by *NEDD9* OE and *SOX9* OE prompted us to examine whether the similar phenomenon occurs in vivo. Following tail vein injection of A375M cells (1 × 10^6^) in NOD/SCID mice, cells treated with the scramble control displayed lung colonization 2 weeks post-injection, whereas no pulmonary metastases were detected from *SOX10* KD and *NEDD9* KD cells (Fig. 6a-d). Moreover, *SOX9* OE at the highest titer (200 μL) exhibited a higher capability than *NEDD9* OE in restoring the metastatic capacity of *SOX10* KD cells (Fig. 6a-d). Consistent with the ability of *SOX9* OE to restore NEDD9 expression in *SOX10* KD cells in vitro, we detected ectopic NEDD9 expression in SOX9 overexpressing cells on the section of lung nodule derived from SOX10 KD + SOX9 OE (Fig. 6e), indicating cell-autonomous induction of NEDD9 by SOX9 OE. These results indicate that increased levels of SOX9 expression can restore the metastatic capacity of *SOX10* KD cells and NEDD9 expression in vivo.

### SOXE and NEDD9 govern migration dynamics of melanoma cells

**Fig. 7:**
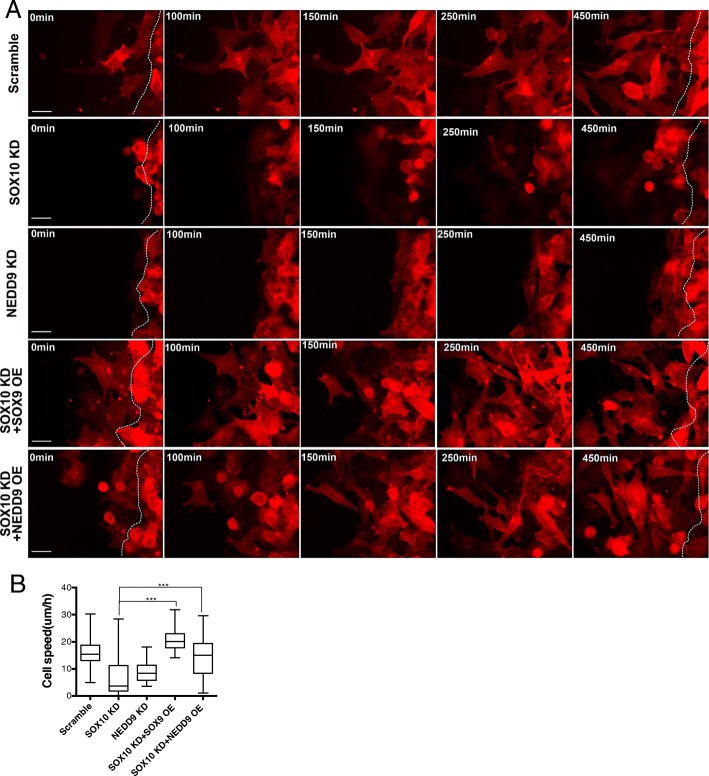
SOXE and NEDD9 direct migration dynamics of melanoma cells. **a** Time-lapse imaging showing the migration dynamics and morphology of A375 cells treated with the indicated constructs and Lifeact-mCherry to mark actin cytoskeleton. White dotted lines indicate the border of the wound. Scale bars: 50 μM. **b** Quantification of the total speed of A375M cells treated with scramble control (*n* = 57), SOX10 KD (*n* = 52), NEDD9 (*n* = 51), SOX10 KD + SOX9 OE (*n* = 67) and SOX10 KD + NEDD9 OE (*n* = 38). Error bars represent mean ± SD of three independent experiments. p*** < 0.001

To evaluate the migratory behavior of melanoma cells transduced with the above 5 different treatments, we performed in vitro time-lapse imaging of wound healing assay for A375 cells, which were transfected with Lifeact-mCherry to label actin cytoskeleton for monitoring real-time morphological change (Fig. 7a). In the 7.5 h period of live cell imaging, we found that cells expressing scrambled shRNA migrated to the wound at an average speed of 15 μM/h and exhibited a mesenchymal mode of migration with membrane protrusions at the cell front (Fig. 7a,b and Additional file 2: Movie S1). Conversely, *SOX10* KD cells were in round shape and acquired amoeboid migration (Additional file 3: Movie S2), while *NEDD9* KD cells tended to migrate in a cluster with elongated morphology (Additional file 4: Movie S3). Both treatments significantly reduced the speed of migration compared to the control (Fig. 7b). However, cells expressing *SOX10* KD + SOX9 OE or *SOX10* KD + NEDD9 OE restored the mesenchymal morphology with migration speed similar to the control (Fig. 7a,b and Additional file 5: Movie S4 and Additional file 6: Movie S5). These data demonstrate that SOXE and NEDD9 are crucial for promoting migration dynamics of melanoma cells.

### SOXE directs mesenchymal-type of melanoma migration through regulation of focal adhesion dynamics and rho GTPases signaling

**Fig. 8:**
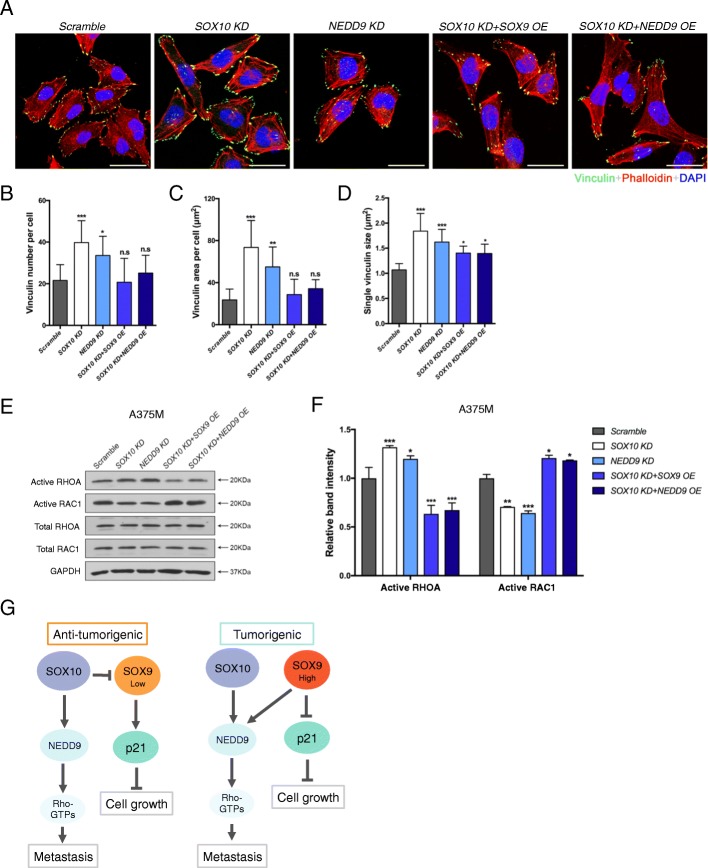
SOXE directs melanoma mesenchymal migration through the NEDD9-mediated focal adhesion dynamics and RHO GTPase signaling. **a** A375M cells treated with the indicated constructs were stained for vinculin and phalloidin. Cell nuclei were counterstained with DAPI. Scale bar: 50 μM. The number of vinculin per cell (**b**), area of vinculin per cell (**c**) and the average size of single vinculin per cell (**d**) were quantified. Thirty cells were analyzed for each treatment. **e** A375M cells treated with the indicated constructs were subjected to RHOA and RAC1 activation assays. GAPDH serves as a loading control. **f** Quantification of band intensity from the densitometric analysis. **g** Schematic model showing a dose-dependent role of SOX9 within a heterogeneous population of melanoma in which low level of SOX9 expression is anti-tumorigenic and high SOX9 is oncogenic. Scale bar: 100 μM. Error bars represent ± SD of three independent experiments. n.s. non-significant, *p < 0.05, **p < 0.01, ***p < 0.001

Previous studies showed that NEDD9 exhibits both positive and negative roles in regulating focal adhesion dynamics and cell motility depending on the cellular context [42, 43] and also contributes to the mesenchymal-type of melanoma migration via modulation of small Rho GTPase activity [29]. Thus, we anticipated that SOX10 or high level of SOX9 regulates NEDD9 expression to promote melanoma migration through alteration of focal adhesion dynamics and RHO signaling activity. To address this issue, we first performed immunofluorescence in A375M cells with five different treatments for vinculin which is one of the key focal adhesion proteins [44] together with phalloidin to mark stress fibers for monitoring cell shape change (Fig. 8a). The dynamic exchange rate (assembly and disassembly) of vinculin will be assessed by quantification of its numbers, area covered and size at the focal adhesion site to indicate positive or negative regulation of cell migration (Fig. 8b-d). The results showed that *SOX10* KD and *NEDD9* KD melanoma cells exhibited an increased number of vinculin^+^ focal adhesions together with larger average area and size per cell compared to the control that could result in enhanced focal adhesion contact with the substratum in vitro and reduced cell motility (Fig. 4d,e, Fig. 7a,b and Fig. 8b-d). In contrast, *SOX9* OE and *NEDD9* OE restored the number; area and size of focal adhesion in *SOX10* KD A375M cells similar to that observed in the control, and rescued their migratory capacity accordingly (Fig. 4d,e, Fig. 7a,b and Fig. 8b-d). It has been well established that RHOA activation is associated with enhanced focal adhesion formation, and the mutual antagonism between RHOA and RAC1 determines cell shape and mode of migration: RHOA-Rho-associated kinase (ROCK) signaling is associated with the amoeboid morphology and RAC1 is associated with the mesenchymal phenotype [30, 45, 46]. In agreement with this, we detected the elevation of RHOA and reduction of RAC1 activities in both *SOX10* KD and *NEDD9* KD cells which are predominantly amoeboid and elongated with relatively less sheet-like protrusions consistent with previous observations in live cell imaging studies (Fig. 7a and Fig. 8a,e,f). This is in contrast to the scramble control, which exhibits a relatively high RAC1 than RHOA activity that is associated with mesenchymal morphology (Fig. 7a and Fig. 8a,e). Conversely, *SOX9* OE and NEDD9 OE led to a reduction of RHOA and upregulation RAC1 activities in *SOX10* KD cells accompanied by acquisition of mesenchymal shape with cytoplasmic extensions (Fig. 7a and Fig. 8a,e,f). These results are consistent with previous findings that NEDD9 is sufficient and required for promoting mesenchymal movement through activation of RAC1 and suppression of RHOA-ROCK driven amoeboid motility [29, 30]. Altogether, our results demonstrate that SOX10 or high level of SOX9 expression could regulate focal adhesion dynamics and Rho GTPase signaling, partly through modulation of NEDD9 activity to promote mesenchymal migration of melanoma.

## Discussion

Because of its neural crest origin, malignant melanoma hijacks a portion of the embryonic neural crest developmental program to initiate their growth and metastasis. In agreement with this notion, transcription factors SOX9 and SOX10 play important roles in neural crest specification and migration [22] but are also involved in melanoma development [18, 47]. SOX10 has been shown to be a crucial regulator in melanomagenesis but previous conflicting reports have not clearly defined whether SOX9 functions as a suppressor or an inducer in melanoma progression [17, 18, 20, 48]. Here we demonstrate that SOX10 is expressed in melanocytic nevus, primary cutaneous, and invasive melanomas where SOX9 exhibits unique but overlapping expression with SOX10. Both SOX9 and SOX10 are co-expressed with pro-metastasis factor NEDD9 to different extents and levels. In agreement with this, SOX10 and/or high SOX9 are required for NEDD9 expression, which is partly responsible for their metastatic properties both in vitro and in vivo. Thus, the levels of the upregulated *SOX9* expression in *SOX10* KD melanoma cell lines are similar to the low mRNA levels of *SOX9* detected in cutaneous melanoma specimens. These levels of *SOX9* expression are able to trigger p21 but not sufficient to induce NEDD9 expression, resulting in suppression of tumor growth and metastasis. This explains why low levels of *SOX9* expression are negatively correlated with *NEDD9* in most primary melanoma specimens. In contrast, further elevation of SOX9 dosage corresponding to high SOX9 in metastatic melanoma specimens lead to opposite effects on p21 and NEDD9 expression with enhanced tumor growth and metastasis as well as induction of MMPs expression. Lastly, SOX10 or high SOX9 regulates focal adhesion turnover and Rho GTPase signaling to promote mesenchymal migration of melanoma cells. Altogether, our studies provide a molecular explanation to reconcile the previous discrepancies that anti-metastatic role of SOX9 is conferred by its sub-optimal level of expression while a high level of SOX9 is pro-metastatic in a heterogeneous population of melanoma (Fig. 8g).

SOX9 has been shown to play an oncogenic role in the formation and growth of tumors in the prostate, the CNS, skin, pancreas, liver, and esophagus [49–53]. However, the previous study by Passeron et al. demonstrated that overexpression of SOX9 in A375 cells inhibited proliferation and tumor growth in xenografts [18]. Similarly, another study by Cheng et al. also showed cell cycle arrest when SOX9 was overexpressed in proliferative melanoma cell lines M010817 and M980513 [20]. Our findings suggest that upregulated *SOX9* expression levels in *SOX10* KD is probably similar to the levels of SOX9 overexpression from these two independent studies and that is sufficient to activate p21 expression for slow growth rate of cells. In addition, we further revealed that elevation of SOX9 expression level resulted in downregulation of p21 and restoration of melanoma proliferation and growth. These data clearly indicate that distinct levels of SOX9 expression impinge on the differential regulation of p21 expression. This dose-dependent effect of SOX9 is also implicated in colorectal cancer model in which a critical dose of SOX9 activity is essential for a maximum rate of proliferation while expression levels higher or lower than this dose would result in the reduction of cell growth [54]. In contrast, we found that overexpression of SOX9 did not have obvious effect on SOX10 expression that differs from a previous report which showed a pronounced downregulation of SOX10 protein by SOX9 overexpression in A375 and M010817 cells [17]. Although the reason for these discrepancies is unclear, it might be caused by using different types of vector (lentiviral vs plasmid) for overexpression that could lead to differential effects of SOX9 on SOX10 expression.

Cheng et al. further showed that overexpression of SOX9 using the same proliferative melanoma cell lines increased their invasiveness to the lungs after intravenous injection [20]. These results are in agreement with our observations that SOX9 overexpression promoted melanoma metastasis in *SOX10* KD both in vitro and in vivo. Furthermore, our immunohistochemistry showed the detection of SOX9 mRNA and protein exclusively in the metastatic melanomas, that is in accord with a previous study in which high SOX9 is associated with lower survival rates of patients with advanced melanoma [20]. The distinct patterns of SOX9 and SOX10 expression in patient specimens probably reflect the heterogeneity of melanoma population harboring different genetic and epigenetic signatures since SOX9 expression could be regulated by DNA methylation [20] and displays antagonistic relationship with SOX10 [17]. Based on our findings together with others, we propose that SOX10 inhibits SOX9 and/or SOX9 promoter is methylated that maintain SOX9 expression at low or sub-optimal level in primary melanoma. As melanoma acquire invasiveness, the promoter of SOX9 becomes hypomethylated probably through downregulation of DNA methyltransferase [20] that could partly contribute to its high level of expression in a subset of SOX10 negative metastatic melanoma.

Although both SOX9 and SOX10 exhibit differential expression patterns in melanomas, whether they share the same or distinct transcriptional targets in mediating the oncogenic events is not known. A previous report revealed that SOX10 transactivates *MIA* expression through its promoter to induce invasive capacity of melanoma cells. Intriguingly, SOX9 activates *MIA* in chondrocytes during mouse chondrogenesis [55]. Likewise, we recently identified *NEDD9* as a direct transcriptional target of SOX9 in mediating avian neural crest delamination [22], whereas the present study suggests that both SOX9 and SOX10 are able to regulate NEDD9 expression partly through transactivating its promoter region. These results suggest that SOX9-regulated developmental genes are being adopted and regulated by SOX10 in controlling the invasive behavior of melanoma. Whether the switching of transcriptional targets occurs only between closely related transcription factors remains to be determined. This could be due to evolutionary change of a few nucleotides within the human gene promoter different from its mouse and chick counterparts that might alter the binding affinity and transactivation capacity between the two closely related transcription factors. In agreement with this notion, our reporter and ChIP assays suggest that the human *NEDD9* promoter sequence favors the binding and transactivation capacity by SOX10 whereas the high level of SOX9 expression is required for efficient activation of NEDD9 expression in both parental and *SOX10* KD melanoma cells. On the other hand, the low level of SOX9 contributes to the p21 activation whereas the high level inhibits its expression. The molecular mechanism underlying the dichotomous role of SOX9 in gene regulation is not known. Given the importance of cofactors in conferring tissue-specific action of SOX9 [56], it is conceivable that distinct SOX9 expression levels may associate with different cofactors to orchestrate differential regulation of target genes and the subsequent impact on melanoma growth and invasion. The identity of these cofactors remains to be discovered by a mass spectrometry-based proteomic method.

Although NEDD9 has been shown to be involved in promoting melanoma metastases [28, 57], the present data showed that it is rather less efficient in restoring pulmonary metastases of SOX10 KD cells in vivo compared to in vitro, probably due to complex in vivo environment that might alter cellular states and responses. In contrast, elevation of SOX9 expression is highly effective in restoring melanoma invasiveness in SOX10 KD cells. This is likely because SOX9 acting upstream of NEDD9 can regulate multiple downstream targets as previously demonstrated by RNAseq analysis, which revealed a few novel candidates that could potentially drive the invasive melanoma phenotype [20]. Indeed, our data showed that high SOX9 not only activates NEDD9 expression to promote mesenchymal migration of melanoma cells through regulation of Rho GTPase activity but also their invasiveness by modulating the expression of various MMPs that mediate extracellular matrix degradation as well as confer immunosuppressive response. Altogether, our findings demonstrate that distinct transcriptional targets of SOX9 at different levels of expression confer melanomas with various cellular properties. It will be worth to perform RNAseq in melanoma cells expressing various levels of SOX9 expression in order to unravel the sets of downstream target genes responsible for the anti-metastatic and the pro-metastatic effects. The outcome of this study would shed new insight into the dosage-dependent transcriptional regulation of SOX9 and also uncover novel druggable targets for the treatment of this devastating disease.

## Conclusions

In conclusion, our findings unravel NEDD9 as a common transcriptional target for SOX10 or high SOX9 to partly mediate their oncogenic features in melanoma, and most importantly reconcile previous discrepancies that low or sub-optimal level of SOX9 expression dictates its anti-metastatic properties whereas high SOX9 is metastatic in a heterogeneous population of melanoma.

## Additional file

Additional file 1: Overexpression of NEDD9 restores the oncogenic properties of *SOX10* KD + *SOX9* KD melanoma cells. (A) AlamarBlue assay for each cell line treated with the indicated constructs. (B) Representative images of crystal violet stained A375M and WM266–4 clones subjected to different treatments. (C) Quantification of the number of A375M and WM266–4 colonies treated with the indicated constructs. (D) Quantification of the number of invaded cells treated with the indicated constructs. (E) DAPI images of transwell invasion of melanoma cells treated with the indicated constructs. Error bars represent ± SD of three independent experiments. *p < 0.05, **p < 0.01, ***p < 0.001. (PDF 619 kb)

Additional file 2: Melanoma cells transduced with scrambled shRNA exhibit mesenchymal-type of movement with membrane protrusions on the front and small-finger like projections in the periphery. This movie file contains 450 min imaging sequences with 5 min intervals between frames, corresponding to the images shown in Fig. 7a. (MOV 10253 kb)

Additional file 3: Melanoma cells transduced with SOX10 KD are round in shape and exhibit amoeboid movement. This movie file contains 450 min imaging sequences with 5 min intervals between frames, corresponding to the images shown in Fig. 7a. (MOV 4770 kb)

Additional file 4: Melanoma cells transduced with NEDD9 KO exhibit elongated morphology and migrate in a cluster. This movie file contains 450 min imaging sequences with 5 min intervals between frames, corresponding to the images shown in Fig. 7a. (MOV 7524 kb)

Additional file 5: Melanoma cells transduced with SOX10 KD + SOX9 OE restore mesenchymal-type of movement with membrane protrusions on the front towards the direction of motion. This movie file contains 450 min imaging sequences with 5 min intervals between frames, corresponding to the images shown in Fig. 7a. (MOV 10444 kb)

Additional file 6: Melanoma cells transduced with SOX10 KD + NEDD9 OE display switching from amoeboid to mesenchymal migration. This movie file contains 450 min imaging sequences with 5 min intervals between frames, corresponding to the images shown in Fig. 7a. (MOV 10208 kb)

## Abbreviations

CAS: a member of the Crk-associated substrate
KD: knockdown
MMP: matrix metalloproteinase
NC: neural crest
NEDD9: Neural precursor expressed, developmentally down-regulated 9
OE: overexpression
SOXE: (Sry (Sex determining gene)-HMG box) E)
