# Supplementary figures and images for: SOX9 is a dose-dependent metastatic fate determinant in melanoma
Source: J Exp Clin Cancer Res. 2019 Jan 14;38:17. doi: 10.1186/s13046-018-0998-6 (PMC6330758; doi:10.1186/s13046-018-0998-6)

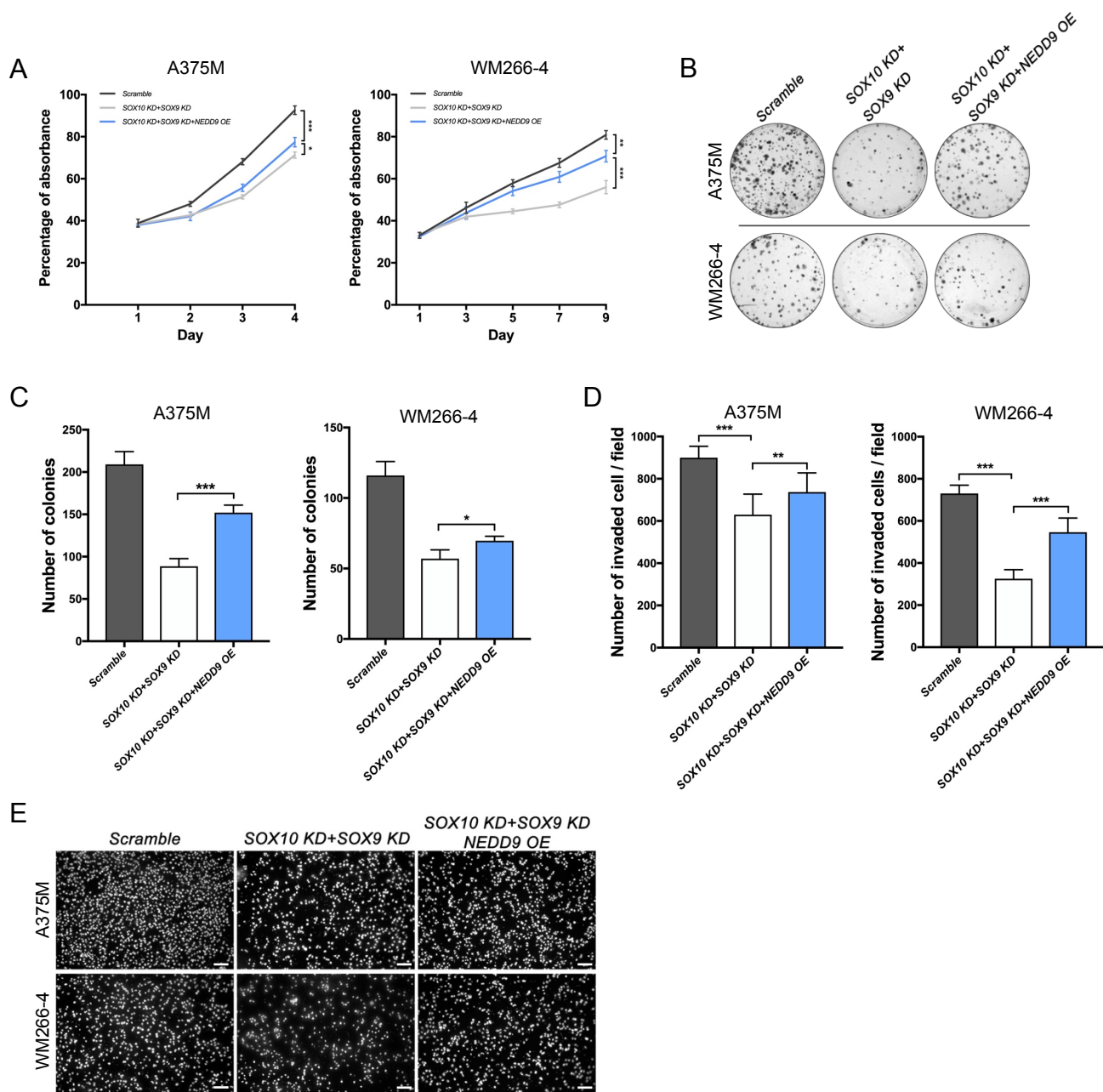

Supplement: Supplementary file 1 — Overexpression of NEDD9 restores the oncogenic properties of SOX10 KD + SOX9 KD melanoma cells. (A) AlamarBlue assay for each cell line treated with the indicated constructs. (B) Representative images of crystal violet stained A375M and WM266–4 clones subjected to different treatments. (C) Quantification of the number of A375M and WM266–4 colonies treated with the indicated constructs. (D) Quantification of the number of invaded cells treated with the indicated constructs. (E) DAPI images of transwell invasion of melanoma cells treated with the indicated constructs. Error bars represent ± SD of three independent experiments. *p < 0.05, **p < 0.01, ***p < 0.001. (PDF 619 kb) [file 13046_2018_998_MOESM1_ESM.pdf]
